# Evaluation of buriti endocarp as lignocellulosic substrate for second generation ethanol production

**DOI:** 10.7717/peerj.5275

**Published:** 2018-08-02

**Authors:** Plínio R. Rodrigues, Mateus F.L. Araújo, Tamarah L. Rocha, Ronnie Von S. Veloso, Lílian A. Pantoja, Alexandre S. Santos

**Affiliations:** 1 Institute of Science and Technology, Federal University of Jequitinhonha and Mucuri Valleys, Diamantina, Minas Gerais, Brazil; 2 Graduate Program in Biofuels, Federal University of Jequitinhonha and Mucuri Valleys, Diamantina, Minas Gerais, Brazil; 3 Department of Basic Sciences, Federal University of Jequitinhonha and Mucuri Valleys, Diamantina, Minas Gerais, Brazil

**Keywords:** Bioethanol, Mauritia flexuosa, Saccharification, Pretreatment

## Abstract

The production of lignocellulosic ethanol is one of the most promising alternatives to fossil fuels; however, this technology still faces many challenges related to the viability of the lignocellulosic alcohol in the market. In this paper the endocarp of buriti fruit was assessed for ethanol production. The fruit endocarp was characterized physically and chemically. Acid and alkaline pre-treatments were optimized by surface response methodology for removal of hemicellulose and lignin from the biomass. Hemicellulose content was reduced by 88% after acid pretreatment. Alkaline pre-treatment reduced the lignin content in the recovered biomass from 11.8% to 4.2% and increased the concentration of the cellulosic fraction to 88.5%. The pre-treated biomass was saccharified by the action of cellulolytic enzymes and, under optimized conditions, was able to produce 110 g of glucose per L of hydrolyzate. Alcoholic fermentation of the enzymatic hydrolyzate performed by *Saccharomyces cerevisiae* resulted in a fermented medium with 4.3% ethanol and a yield of product per substrate (Y_P/S_) of 0.33.

## Introduction

The growth of human population, depletion of fossil fuels and rising concerns about the anthropic impact on the environment have encouraged the search for renewable sources for cleaner energy production (Severo, Guimarães & Dorion, 2018; Sarkar et al., 2012). In this context, lignocellulosic biomasses are a promising feedstock for liquid biofuels syntheses, alternative to petroleum-based fuels (Saini, Saini & Tewari, 2015).

The technology for the production of second generation (2G) bioethanol, or lignocellulosic ethanol, has evolved in the last decades and functioning industrial plants already exist in some parts of the world, nevertheless; this biofuel still faces challenges of feedstock access, supply chain infrastructure, and price competitiveness with petroleum industry (UNCTAD, 2016).

Lignocellulosic ethanol can be obtained from fermentation of hexose and pentose derived from the polysaccharides constituting plants cell walls and requires additional operations to those normally applied to produce first generation ethanol (Zabed et al., 2016). Lignin removal and hemicellulose hydrolysis, followed by cellulose saccharification, are necessary steps to provide fermentable sugars offered to specialized microorganisms to produce 2G ethanol (Maurya, Singla & Negi, 2015; Guo, Chang & Lee, 2018). There is a large set of biomass being evaluated as raw materials for this nascent industry, with emphasis on agro-industrial residues (Macedo et al., 2011; Hoa, Ngob & Guo, 2014; Domínguez-Bocanegra, Torres-Muñoz & López, 2015).

Buritizeiro (*Mauritia Flexuosa*) is one of the most abundant species of palm tree in Brazil, its occurrence covers the Cerrado and Amazon national biomes. Its fruit (Buriti) is elliptical to oval in shape and comprised of pericarp (bark), mesocarp (pulp), endocarp (seed shell lignocellulosic tissue), and endosperm (seed) (Sampaio & Carrazza, 2012; Virapongse et al., 2017). Buriti fruits are economically exploited for a variety of purposes, such as edible and cosmetic oil extraction, and manufacturing of beverages, flours and ice creams (Lorenzi et al., 2004; Manzi & Coomes, 2009; Gilmore, Endress & Horn, 2013).

The market demand for the fruit is growing (Virapongse et al., 2017), however; the endocarp fruit portion presents few alternatives for commercial use, having low economic value. Buriti fruit production in north and northeast of Brazil, main regions of production in the country, is estimated at 70,000 tons per harvest (Bovi, 2015), and its endocarp is a possible source for the production of 2G ethanol, since it is an abundant waste of buritizeiro palm exploitation and does not directly require the availability of more cultivation lands (Van, Brose & Schenkel, 2011; Bos et al., 2016).

Also, buriti endocarp use does not present competition with food market chains and embodies potential income generation, representing an alternative for communities living from the buriti fruit to access cleaner energy sources and to achieve a certain degree of energy independence.

In this paper, buriti fruit endocarp was physically and chemically characterized and evaluated for 2G ethanol production. Pre-treatments with dilute sulfuric acid and sodium hydroxide, and enzymatic saccharification were performed using response surface methodology. The effects of the factors studied in the pre-treatment and in the enzymatic hydrolysis were evaluated and the optimal conditions were highlighted. Ultimately, saccharified cellulose from buriti endocarp was fermented to ethanol using *Saccharomyces cerevisiae.*

Cellulose fraction of the buriti endocarp is surrounded by a matrix of hemicellulose and lignin, which together promote steric hindrance on the cellulose saccharification process. Chemical treatments, such as dilute acid and alkaline hydrolysis, are considered to be the most suitable for the commercial scale hemicellulose and lignin removal, increasing exposure of cellulase target sites on cellulose (Sims et al., 2009; Aditiya et al., 2016).

## Material and Methods

### Physical characterization of buriti fruits

**Figure 1:**
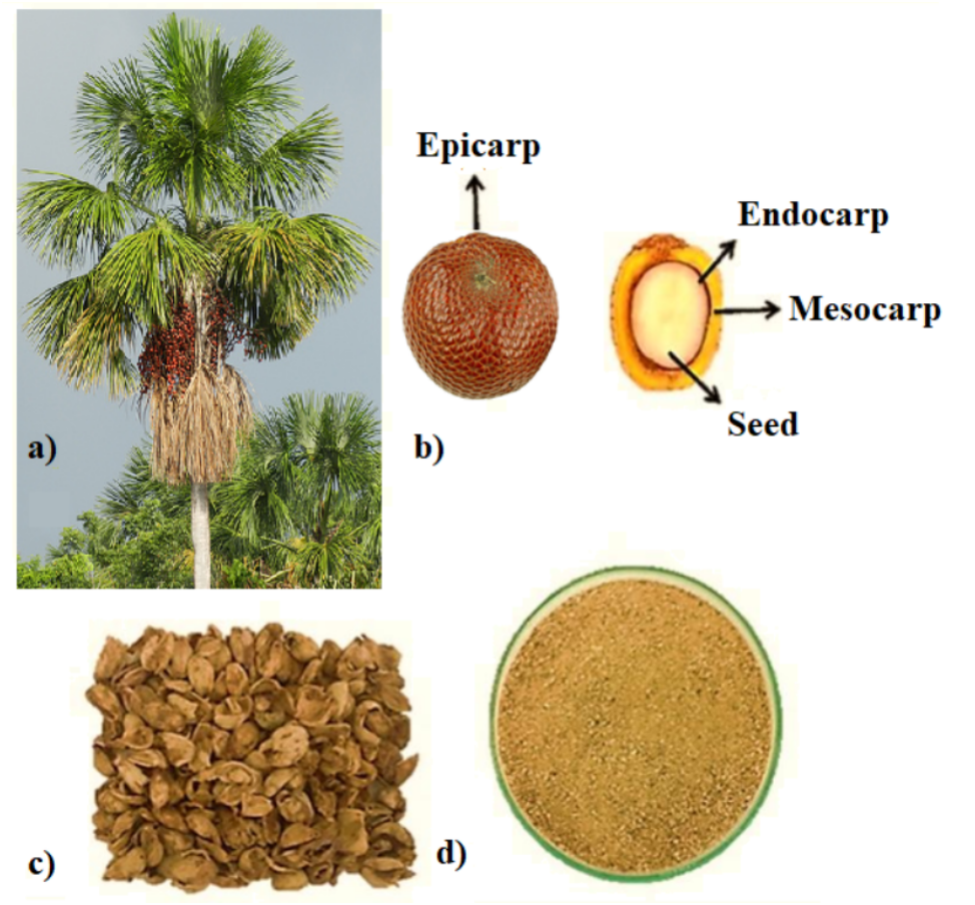
Appearance of *Mauritia flexuosa* L palm tree (A), its whole and sectioned fruits, and their (B), dry (C) and ground endocarps (D). Photo credit: Plínio R. Rodrigues

Twenty kilograms of fruits were collected in Três Marias city, in Minas Gerais, Brazil. The physical characterization was performed on 50 randomly selected fruits. The fruits were weighed in the analytical balance, with an accuracy of 0.1 mg, and their longitudinal and transversal diameters were measured with the aid of a digital caliper, with an accuracy of 0.02 mm. Results were expressed with two decimals. The endocarp of each fruit was separated manually with the aid of a steel knife (Tramontina) and weighed. After separation, the endocarps were oven dried with forced air ventilation at 60 °C for 24 h, ground with a manual grinder (B03-Botini), manually sieved for particle size standardization, between 40 and 20 mesh (0.42 to 0.84 mm), and stored in polyethylene bags at room temperature, protected from light (Fig. 1).

### Chemical characterization of buriti fruit endocarp

The ground endocarps were characterized in terms of total moisture, ashes, proteins, lipids, crude fiber, starch, total soluble sugars (TSS), cellulose, hemicellulose and lignin contents. All experiments were executed in three replicates.

#### Moisture

A total of 3.0 g of endocarp samples were weighed in glass plates, previously dehumidified in an oven at 105 °C, and kept in an oven (TE-394/2; Tecnal, Ourinhos, SP, Brazil) at 105 °C until constant weight. The result was calculated gravimetrically according to Adolph Lutz Institute (IAL, 2008).

#### Ashes

Ash content was determined, according to IAL (2008), from 0.5 g of endocarp samples contained in porcelain crucible, previously calcined in muffle at 550 °C. The samples were previously carbonized and then incinerated in muffle at 550 °C. After complete incineration, the crucibles containing the samples were cooled in a desiccator and weighed.

#### Proteins

Protein content was quantified by the Kjeldahl method, according to IAL (2008), adding 0.5 g of sample, 600 mg of potassium sulphate, 10 mL of concentrated sulfuric acid and 300 mg of copper sulphate in a glass tube, followed by digestion at 350 °C. Subsequently, 50 ml of 50% sodium hydroxide was added to the tubes and the mixture was distilled in nitrogen distiller (Tecnal, Ourinhos, SP, Brazil). The distillate was collected in a 250 mL conical flask containing 15 mL of saturated boric acid solution and methyl red indicator solution (0.1%). Subsequently, the distillate was titrated with 0.02N of hydrochloric acid.

#### Lipids

Endocarp samples (1.0 g) were transferred to qualitative filter paper, with a porosity of 12.5 µm, and placed in Soxhlet extractor. Lipids were extracted with ethyl ether approximately for 8 h and collected in 250 mL flat bottom dehumidified flasks. After extraction, the flasks were placed in the oven at 105 °C for solvent evaporation and lipids quantification (IAL, 2008).

#### Crude fiber

Crude fiber content was determined with the addition of 0.5 g of the sample, 17.5 mL of 70% acetic acid and 0.5 g of trichloroacetic acid in test tubes, which were digested at 110 °C for 30 min. Subsequently, the material was filtered using pre-sintered filters, containing glass wool, washed with distilled water (95 ± 2 °C), until pH neutrality and oven dried at 105 °C (Kamer & Ginkel, 1952).

#### Starch and total soluble sugars (TSS)

Starch and total soluble sugars were determined according to the methodology described by Mccready et al. (1950). The quantification was performed with anthrone 0.1% in sulfuric acid addition and absorbance determination in the spectrophotometer.

#### Cellulose, hemicellulose, and lignin

Cellulose, hemicellulose and lignin contents were quantified by neutral detergent fiber (NDF) and acid detergent fiber (FDA) methods described by Van Soest (1963), Van Soest (1964) and Van Soest (1968).

### Pre-treatment of buriti endocarp

Whole buriti endocarps, dried and ground, were pre-treated with dilute sulfuric acid followed by hydrolysis with alkali to remove fractions of hemicellulose and lignin, respectively. The experiments were executed in three replicates.

#### Treatment with dilute sulfuric acid

Determination of optimal conditions for acid pre-treatment of the biomass was accomplished through the use of a Rotational Central Composite Design (RCCD) that evaluated the influence of reaction time; 20 min (−1) and 60 min (−1), solid–liquid (S/L) ratio; 10% (−1) and 20% (+1), and concentration of sulfuric acid (H_2_SO_4_); 2% (−1) and 7% (+1), on the removal of hemicellulose contained in the buriti endocarp. In each test, carried out in a glass tube (30 × 2.5 cm), 1 g of dry sample was added with the solution of H_2_SO_4_ in the pre-defined concentration and proportion for each of the 18 tests generated by the factorial matrix 2^3^, containing four central points and six axial points. The tests were performed in an autoclave at a fixed temperature of 120 °C. Treatment conditions and parameters were based on Corbin et al. (2015) work.

#### Determination of sugars removed by acid pre-treatment

Quantification of glucose released after acid hydrolysis was determined by the enzymatic-colorimetric method described by Lloyd & Whelan (1969). Quantification of reducing sugars (RS) in the acid hydrolyzate was carried out using the dinitrosalicylic acid method described by Miller (1959). The decomposition of hemicellulose was expressed in grams of sugar released per 100 g of biomass.

#### Pre-treatment with sodium hydroxide

Optimization of lignin removal from buriti endocarps was performed by 2^2^ RCCD factorial experiments, which investigated the influence of process temperature; 30 °C (−1) and 80 °C (+1), and concentration of sodium hydroxide (NaOH ); 2% (−1) and 12% (+1), in addition to four central points and four axial points. In each test, carried out in the glass tube (30 × 2.5 cm), 1 g of dry sample, sodium hydroxide solution at a solid–liquid ratio of 10%, were added and then incubated in a water bath for the period of 12, 24, 36 and 48 h. Lignin removal was estimated by total phenolic compounds dosage using gallic acid as standard, according to Singleton & Rossi (1965).

### Enzymatic saccharification of pretreated endocarp

For the saccharification process optimization, an RCCD with three factors was performed. Analyzing the effect of S/L ratio; 5% (−1) and 15% (+1), time; 6 h (−1) and 24 h (+1), and enzyme concentration (Celluclast; Novozymes, Bagsværd. Denmark); 20 µL g^−1^ (−1) and 100 µL g^−1^ (+1), with four central points and six axial points. In each condition described by the RCCD planning, the mass of 1 g of pre-treated endocarp was used in a 50 mL conical flask, followed by the addition of 50 mM sodium bicarbonate buffer (pH 5.0) and enzyme volume according to experimental planning. Pre-treated endocarp was rinsed with distillate water and dried before utilization. The tests were incubated at 50 °C with agitation of 100 rpm. At the end of each reaction, the concentrations of glucose and reducing sugars (RS) were determined in the soluble fraction of the hydrolyzate. The digestion of cellulose was expressed in grams of glucose released per 100 g of biomass.

### Alcoholic fermentation of the enzymatic hydrolyzate

Fermentation of the optimized enzymatic hydrolyzate was carried out in 250 mL conical flasks coupled to fermentometers, a glass system that allows carbon dioxide (CO_2_) release and prevents the entry of external air, at room temperature (25 ± 2 °C). Dehydrated commercial baker’s yeast (Fleischmann^®^) of the *Saccharomyces cerevisiae* species was used as a fermentation agent in the ratio of 1% (w/v) to the must volume. No nutrient supplementation was applied. The fermentative process was monitored gravimetrically for CO_2_ release. The measurement of the mass of gas released was used to estimate the ethanol production and fermentable sugars consumption at every two hours until the end of the fermentation. Ethanol concentration was quantified by the potassium dichromate method (Isarankura-Na-Ayudhya et al., 2007). Glucose and reducing sugars (RS) concentrations were also determined.

### Statistical analysis

Modeling, graphing and analysis of the results obtained with the rotational central composite designs were performed using tools available in Statistica 8.0 software (Statsoft Inc., Tulsa). ANOVA with *p* < 0.05 level was stipulated as the statistical parameter of significance.

## Results

Physical characterizations of fresh buriti fruits are displayed in Table 1, the values are presented in percentage averages followed by their standard deviations. Integral fruits presented an average mass of 38.33 ± 9.06 g, the transversal diameter of 38.75 ± 3.76 mm and longitudinal diameter of 48.68 ± 2.94 mm (Table 1). The endocarp mass represented, on average, 25.3% of the whole fruit.

**Table 1:** Physical characterization of *in natura* buriti fruit.

Parameter	Average
Pulp mass (g)	9.26 ± 2.94
Epicarp mass (g)	9.40 ± 2.04
Endocarp mass (g)	9.72 ± 3.00
Peduncle mass (g)	0.83 ± 0.26
Seed mass (g)	6.01 ± 2.16
Transversal diameter (mm)	38.75 ± 3.76
Longitudinal diameter (mm)	48.68 ± 2.94

Table 2 shows the Buriti fruit endocarp chemical composition. It is possible to observe a crude fiber content greater than 26% and four main sugar sources that could be converted to bioethanol; starch, cellulose, hemicellulose and soluble sugars. Total carbohydrates portion corresponded to 44.2% of the biomass (Table 2). Cellulose was the main polymer on the endocarp composition, with a content of 22.15%, hemicellulose fraction corresponded to 10.73 %, and lignin fraction was 11.79%. Moisture, lipids, ash and protein contents are also listed in Table 2.

**Table 2:** Chemical characterization of buriti fruit endocarp. Percentage averages followed by their standard deviations.

Composition	Endocarp (Seed shell)
Moisture (%)	9.54 ± 0.19
Ash (%)	4.46 ± 0.08
Lipids (%)	4.39 ± 0.37
Total Proteins (%)	3.62 ± 0.08
Crude Fiber (%)	26.37 ± 0.44
TSS (%)	4.49 ± 0.13
Starch (%)	6.80 ± 0.18
Cellulose (%)	22.15 ± 2.43
Hemicellulose (%)	10.73 ± 0.79
Lignin (%)	11.79 ± 0.30

**Notes.**

TSS Total Soluble Sugars

In this study, buriti endocarp was subjected to a sequence of acid and alkali treatments with the purpose of exposing the cellulose polymers to the enzymatic hydrolysis to obtain monomers of hexoses for their subsequent anaerobic fermentation by *S. cerevisiae* yeast.

The quantities of reducing sugars and glucose removed per 100 g of endocarp subjected to acid pretreatment under the experimental design conditions are shown in Table 3. There was the greater release of reducing sugars in the condition of test 10 (15% S/L ratio, 8.04% H_2_SO, 40 min). In this point, 8.75 g of reducing sugars per 100 g of biomass was removed. On the other hand, in the condition of test 5 (10% S/L ratio, 7% H_2_SO, 20 min), 8.02 g of reducing sugars per 100 g of biomass were removed, 9% less than under test condition 10. Test 5 was then chosen as the optimal condition for the preparative test for using less acid concentration, presenting great RS release (hemicellulose removal) and half the reaction time of test 10.

**Table 3:** Rotational central composite design for the acid pretreatment of buriti endocarp (1 atm, 120°C) with its respective response factors.

Test	Independent variables			Response factors	
	S/L ratio (%)	H_2_SO_4_ (%)	Time (min.)	Glucose (%)	RS (%)
1	10	2.00	20.0	0.92	2.22
2	20	2.00	20.0	0.38	0.85
3	10	2.00	60.0	1.52	5.63
4	20	2.00	60.0	0.70	2.55
5	10	7.00	20.0	1.84	8.02
6	20	7.00	20.0	1.05	5.01
7	10	7.00	60.0	0.79	4.26
8	20	7.00	60.0	0.35	2.52
9	15	0.96	40.0	0.13	0.90
10	15	8.04	40.0	2.10	8.75
11	15	4.50	11.7	0.57	3.21
12	15	4.50	68.3	1.68	7.30
13	7	4.50	40.0	1.21	5.10
14	22	4.50	40.0	0.92	4.70
15	15	4.50	40.0	1.46	6.65
16	15	4.50	40.0	1.11	5.90
17	15	4.50	40.0	1.37	6.71
18	15	4.50	40.0	1.24	6.88

**Notes.**

S/L Solid-liquid ratio RS reducing sugars

The concentration of H_2_SO_4_ had a significant and positive linear effect (*p* < 0.05) on hemicellulose removal and the interaction of H_2_SO_4_ concentration over time (*p* < 0.05) had a negative effect. Negative quadratic individual effects of H_2_SO_4_ and S/L ratio were also observed, however, with *p* values of 0.114 and 0.102, respectively.

**Figure 2:**
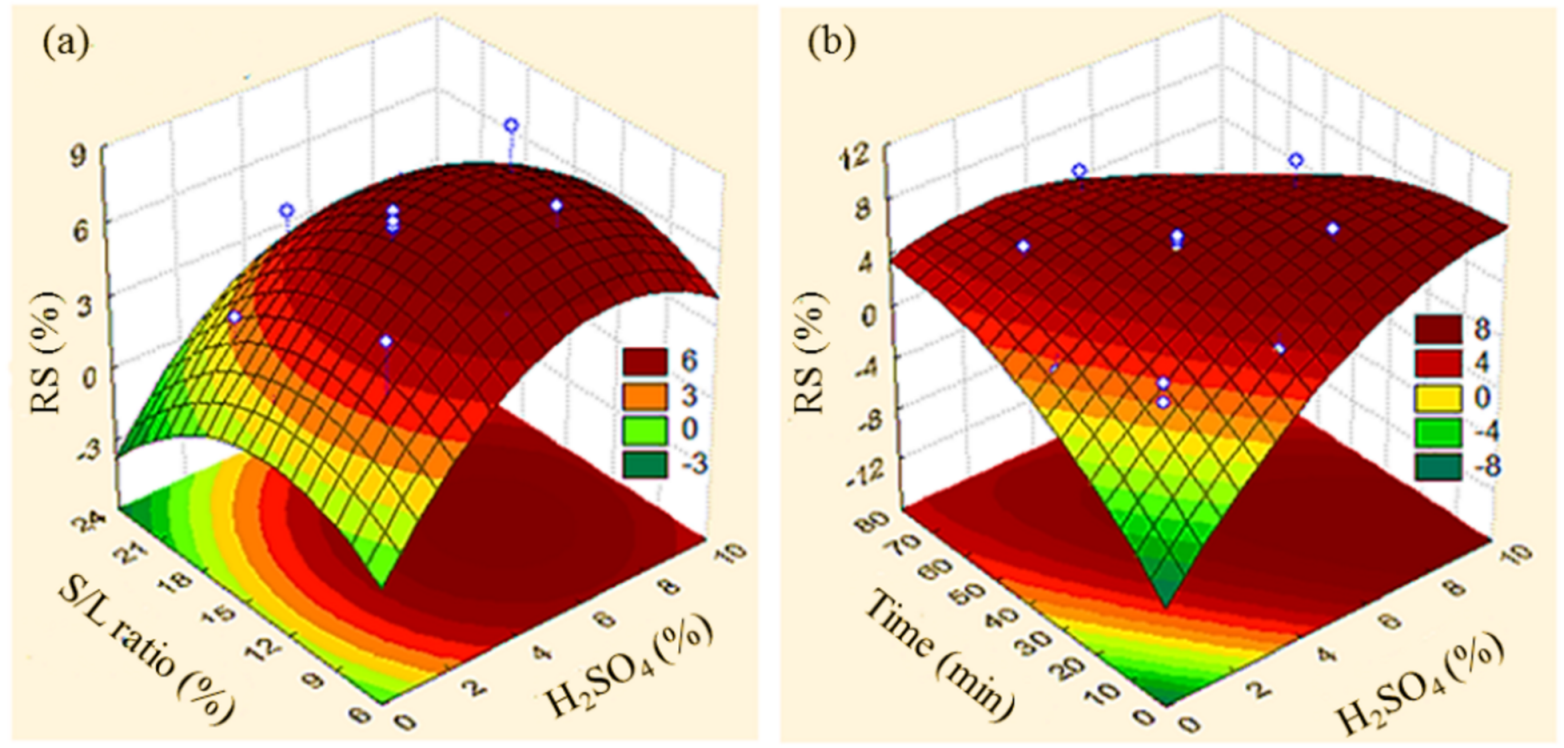
Response surface graphs. Percentage of reducing sugars (RS) removed in the acid hydrolysis of the buriti endocarp as a function of the combined effects of the (A) concentration of H_2_SO_4_ with solid–liquid ratio (S/L), and (B) time with H_2_SO_4_ concentration.

The combined effects of H_2_SO_4_ concentration with the S/L ratio factors and H_2_SO_4_ concentration over time on the removal of the hemicellulosic portion from the buriti endocarp are presented as response surface curves in Fig. 2; the coefficient of determination (*R*^2^) was 0.81. Not coincidentally, the areas under the response surface curves (Fig. 2) representing the regions with the highest hemicellulose removal refer to the combination of factors indicated by the conditions of test 5 (Table 3).

Characterization of the lignocellulosic fraction of buriti endocarp recovered after acid pretreatment (51.4% mass recovery), using the optimum condition defined, indicated changes in the lignocellulosic content. Cellulose, hemicellulose and lignin fractions found were, respectively; 43.07 ± 0.47%, 1.29 ±0.04% and 24.50 ± 0.38%. It is possible to see the great impact of the treatment on hemicellulose removal, which showed an 88.26% reduction on content, compared to the raw endocarps, followed by concomitant concentration of the cellulose and lignin fractions, polymers that were not removed by acid action.

The results for the RCCD of lignin removal from the acid pre-treated buriti endocarps are presented in Table 4 for times of 12, 24, 36 and 48 h. In all caustic hydrolysis times evaluated, the alkali used (NaOH) had a positive and significant effect (*p* < 0.05) on lignin removal. The temperature also had a positive and significant effect (*p* < 0.05) on lignin removal at all evaluated times. The regression analysis squared correlation coefficients (R^2^) were 0.97, 0.83, 0.94 and 0.93, for the times of 12, 24, 36 and 48 h, respectively.

**Table 4:** Rotational central composite design for the alkaline pretreatment of the buriti endocarp remaining from the acid pretreatment with its respective response factor in the times of 12 h, 24 h, 36 h and 48 h.

Test	Independent variables		Total phenolic compounds			
	NaOH (%)	Temperature (°C)	12 h (%)	24 h (%)	36 h (%)	48 h (%)
1	2.00	30.00	0.86	1.56	1.93	2.33
2	2.00	80.00	1.73	2.31	3.75	4.45
3	12.00	30.00	1.88	1.96	2.33	3.28
4	12.00	80.00	3.28	5.69	8.40	9.64
5	0.95	55.00	0.48	1.06	1.23	1.85
6	13.05	55.00	2.43	2.96	4.65	4.84
7	7.00	24.75	1.11	1.43	2.06	3.75
8	7.00	85.25	3.09	3.92	5.69	6.55
9	7.00	55.00	1.77	2.54	4.87	5.28
10	7.00	55.00	1.83	2.57	4.91	5.15
11	7.00	55.00	1.81	2.49	4.82	5.25
12	7.00	55.00	1.75	2.43	4.89	5.23

As can be seen in Table 4, the reaction time of 48 h was the most favorable for lignin removal. The polynomial fit that describes the percentage of lignin removal, expressed in the form of phenolic compounds, as a function of the temperature and NaOH concentration in the time of maximum lignin hydrolysis (48h) is represented by Eq. (1).

(1) \begin{eqnarray*}\mathrm{L}=5.10+2.82\mathrm{C}-1.75{\mathrm{C}}^{2}+3.43\mathrm{T}+0.71{\mathrm{T}}^{2}+2.12\mathrm{CT}\end{eqnarray*}

Where:

*L* = Total phenolic compounds (%);

*C* = NaOH concentration (%) (m/v);

*T* = Temperature (°C).

**Figure 3:**
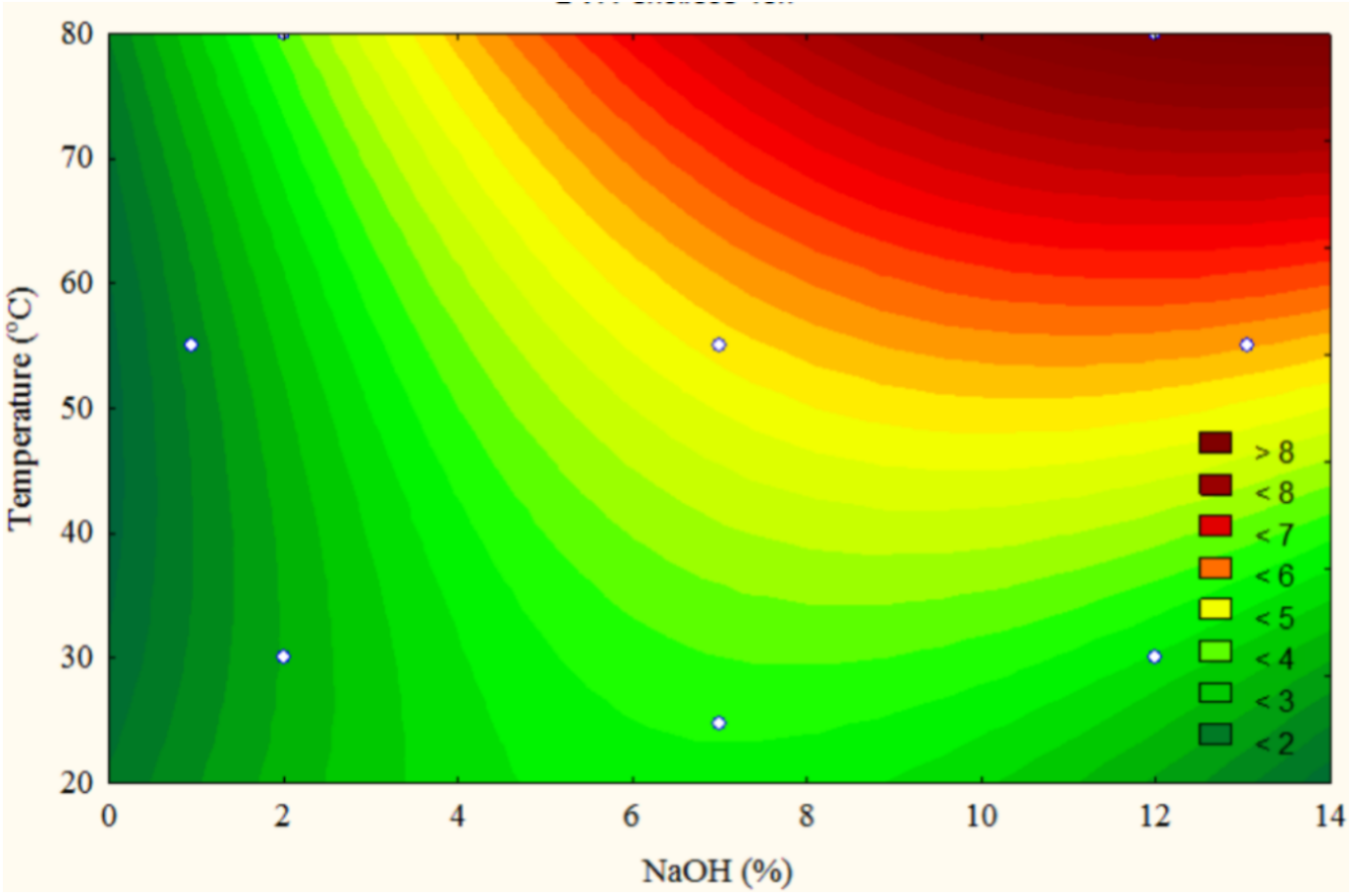
Projection of the adjusted response surface curve for the rotational central composite design in the time of 48 h.

The graph of the projection of response surface to the time of 48 h is seen in Fig. 3. The region where the maximum release of phenolic compounds can be observed is represented by a temperature higher than 80 °C and NaOH concentration between 10 and 14%. The highest release of phenolic compounds was observed in the reaction medium with 12% NaOH and temperature of 80 °C (test 4, Table 4). This condition was adopted for the preparatory pretreatment of the biomass previously submitted to acid pretreatment.

Characterization of the lignocellulosic fraction of the biomass recovered after the alkaline pre-treatment (49.5% mass recovery) showed that there was a 64% reduction in lignin concentration when compared to the raw biomass (untreated) and 83% when compared to the biomass after the acid pretreatment. The contents of cellulose, hemicellulose, and lignin in the endocarp after caustic hydrolysis were; 88.54 ± 0.38%, 2.73 ± 0.16% and 4.20 ± 0.28%, correspondingly.

The experiments to optimize the saccharification process of cellulose contained in the pretreated biomass are displayed in Table 5. The condition of the test that presented the greatest release of glucose (in average 77.08%) or reducing sugars (in average 80.65%) was described by the central points, tests 15, 16, 17 and 18 (60.00 µL g^−1^ cellulase, 10.00% S/L ratio and 15.0 h).

**Table 5:** Rotational central composite design used for the enzymatic saccharification of the buriti endocarp sequentially pretreated with acid and alkali and their respective response factors.

Test	Cellulase (µL g^−1^)	S/L ratio (%)	Time (h)	Glucose (%)	RS (%)
1	20.00	5.00	6.0	11.36	14.09
2	20.00	5.00	24.0	16.60	16.81
3	20.00	15.00	6.0	17.83	16.23
4	20.00	15.00	24.0	51.39	56.06
5	100.00	5.00	6.0	23.60	21.13
6	100.00	5.00	24.0	33.21	31.12
7	100.00	15.00	6.0	46.67	46.95
8	100.00	15.00	24.0	61.20	59.24
9	3.43	10.00	15.0	6.99	5.67
10	116.57	10.00	15.0	53.49	58.72
11	60.00	2.93	15.0	20.97	19.69
12	60.00	17.07	15.0	31.46	32.91
13	60.00	10.00	2.3	10.48	15.20
14	60.00	10.00	27.7	63.63	61.77
15	60.00	10.00	15.0	76.92	80.59
16	60.00	10.00	15.0	76.04	79.99
17	60.00	10.00	15.0	78.67	80.35
18	60.00	10.00	15.0	76.04	81.68

**Figure 4:**
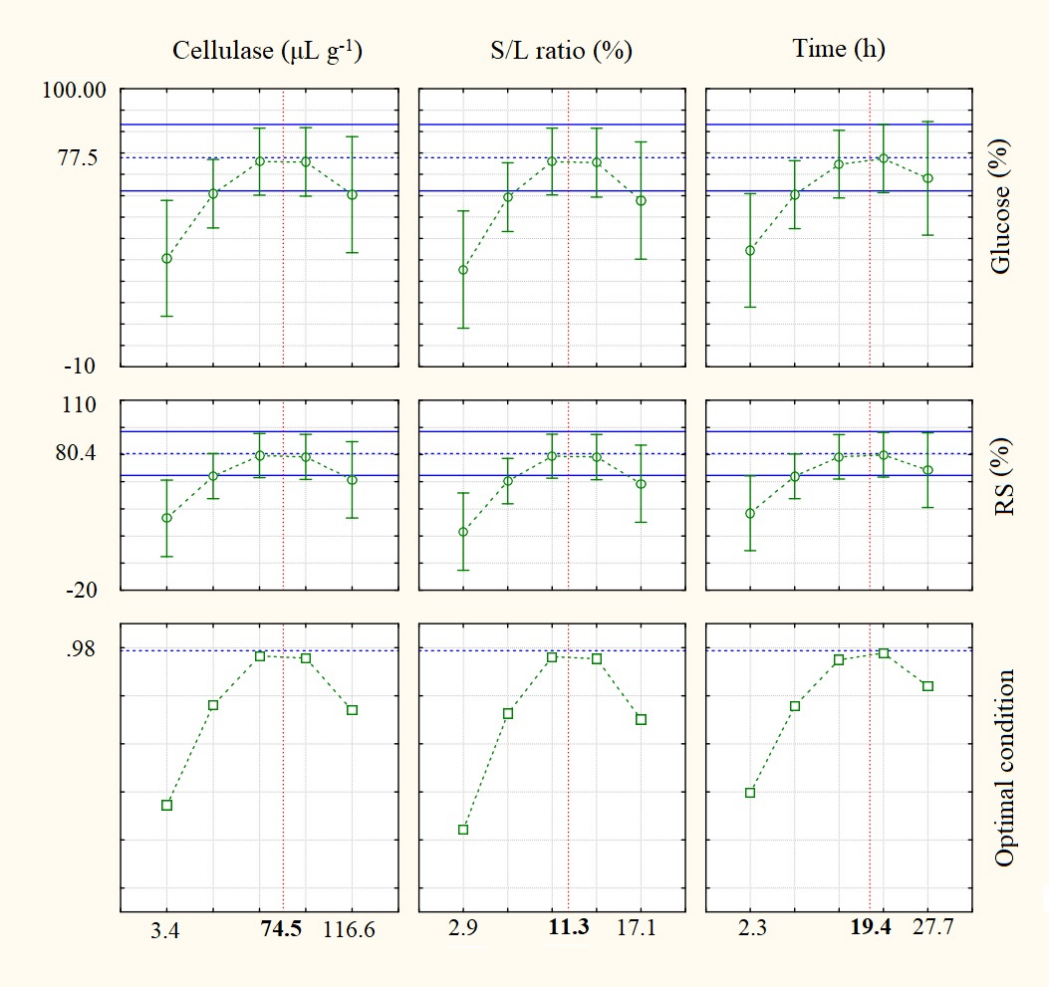
Profile of predicted and desirable glucose and reducing sugar (RS) values. (*y*-axes) for cellulase concentration, S/L ratio and process time factors (*x*-axes) used in saccharification of the cellulose contained in the pre-treated buriti endocarp.

All quadratic effects were negative and significant (*p* < 0.05), indicating that there were maximum points in the hydrolytic phenomenon. The release profiles of glucose and reducing sugars under the conditions of the RCCD can be seen in Fig. 4, as well as the optimal condition highlighted for saccharification of the biomass. The model obtained by the experimental design had a regression coefficient (*R*^2^) of 84.15% for the glucose release and 84.42% for the release of reducing sugars. The optimum condition indicated by the response surface methodology (Fig. 4) showed the combined use of 74.50 µL of Cellulase g^−1^ of biomass, 11.30% for the S/L ratio and 19.40 h of reaction time.

After applying the optimum saccharification conditions in a preparative test with the pre-treated buriti endocarp, the hydrolyzate obtained contained 129.68 ± 0.72 g L^−1^ of reducing sugars and 110.14 ± 0.63 g L^−1^ of glucose, displaying and hydrolytic efficiency of 86.16%.

**Figure 5:**
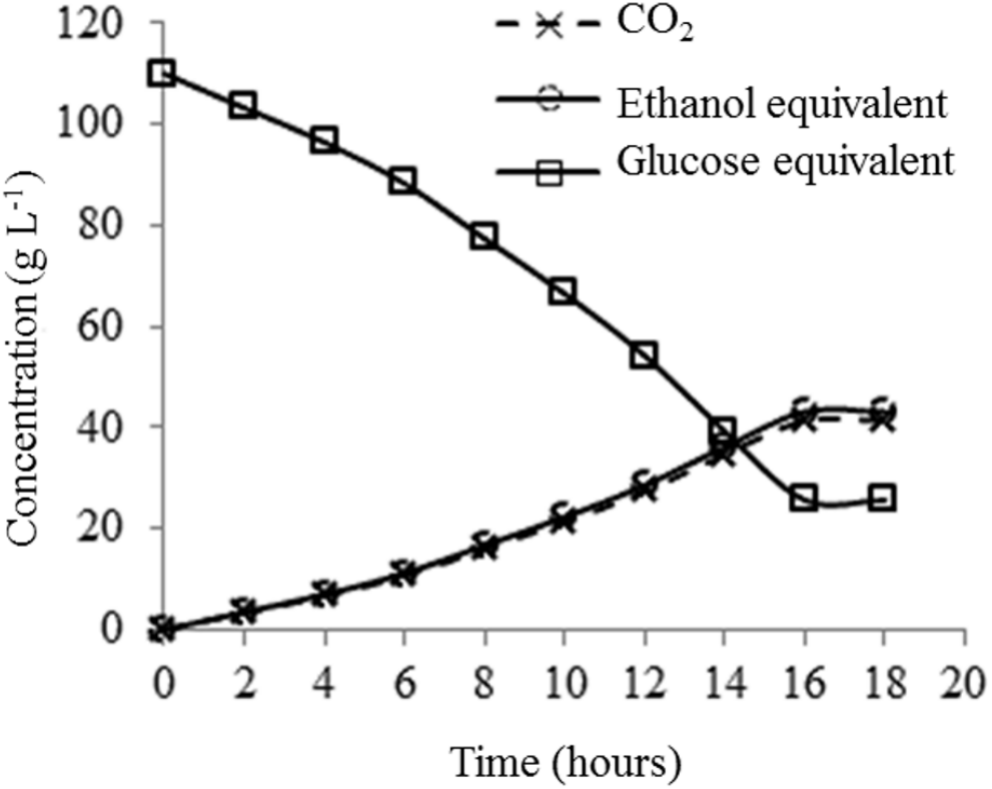
Progress of the alcoholic fermentation of the enzymatic hydrolyzate of the pre-treated buriti endocarp, conducted in anaerobiosis with *Saccharomyces cerevisiae* yeast.

The fermentation process was monitored gravimetrically by the evolution of CO_2_ and the equivalent values of glucose consumed and ethanol produced was calculated during 18 h of reaction (Fig. 5). At 16 h the fermentation had been completed since no change in the mass of the fermentative system was observed.

Once the fermentation was complete, the system was opened and the glucose, reducing sugars and ethanol contents were determined analytically. Thus, 43.16 g L^−1^ of ethanol was produced, with a fermentative efficiency (ethanol yield) of 77% or 0.33 g EtOH g RS^−1^. The concentrations of glucose and RS at the end of fermentation were, respectively; 25.10 g  L^−1^ and 37.59 g L^−1^.

## Discussion

Palm trees endocarp composition data are scarce in literature, what highlights the relevance of Table 2 information. Comparatively, Saka et al. (2008) found a similar cellulose content (20.5%) on *Elaeis guineensis* endocarp to that found for *Mauritia flexuosa* endocarp in the present study, nonetheless the hemicellulose content reported by the cited authors was two times higher (22.3%) than that described in this paper. Is important to mention that chemical composition of a biomass is mainly determined by its evolutionary history and varies significantly with the species (Mendu et al., 2011; Dardick & Callahan, 2014). The lignocellulosic fraction also varies depending on the stage of development and the anatomical part of the plant (Handley, Pharr & McFeeters, 1983; Dardick & Callahan, 2014).

Regarding the dilute acid treatment described in the present study, the concentration of H_2_SO_4_ had a significant positive linear effect on hemicellulose removal. A negative effect on the interaction of H_2_SO_4_ concentration over time was also noted. Probably, such phenomenon is due to dehydration of glucose to hydroxymethylfurfural promoted by the acid at the longest reaction times ( Deshavath et al., 2017; Woo et al., 2015). In addition, it is also probable that the glucose found in the hydrolyzate was the product of the hydrolysis of starch present in the endocarp since β 1,4 glycosidic bonds between glucose residues of cellulose are recalcitrant to the action of dilute acids.

In their studies of dilute acid pre-treatment (1 h, 121 °C, 0.5 M H_2_SO_4_) of red and white grape marcs, Corbin et al. (2015) achieved 58% of total carbohydrates liberation from the red marc and 84% from the white marc. Gonzales, Sivagurunathan & Kim (2016) reported a hemicellulose recovery of 57.4% from empty palm fruit bunch (60 min, 121 °C, 5% H_2_SO_4_). Zhang et al. (2011), in turn, reported 74.5% release of the total hemicellulose in his pretreatment assessments of cattails (*Typha* species) (15 min, 180 °C, 1% H_2_SO_4_), demonstrating inferior performances than what was accomplished in this paper.

After caustic hydrolysis, the buriti fruit endocarp, previously treated with dilute acid, showed a drastic reduction of lignin content compared to the raw biomass. Barliante et al. (2015) published a lignin removal of 47.3% from oil palm fronds with alkaline hydrolysis as the sole treatment in their best conditions set (30 min, 150 °C, 10% NaOH). Chieng et al. (2017) reported 78.7% decrease in lignin content of oil palm mesocarp fiber, after sequential alkaline (3 h, 80 °C, 4% NaOH) and dilute acid (45 min, 45 °C, 65% H_2_SO_4_) treatments. Although the last authors achieved a hydrolysis performance less efficient than what is described in the present paper, they attained a similar final lignin fraction in their biomass than that revealed in the present study.

Also applying sequential alkaline and diluted acid hydrolysis, but using calcium hydroxide and peracetic acid, Azelee et al. (2014) reported 59.25% lignin removal (1 g L^−1^ Ca(OH)_2_, S-L ratio of 1:8, 50 °C for 1.5 h; followed by 20% peracetic acid pretreatment at 75 °C for 2 h) in their studies of ethanol production from kenaf (*Hibiscus cannabinus*).

Enzymatic saccharification performed revealed negative quadratic effects indicating maximum points of hydrolytic efficiency, probably due to the exhaustion of the susceptible substrate and/or limitation in mass transfer, which can largely affect the enzymatic attack. The yield of glucose in the optimum condition (60.00 µL g^−1^ cellulase, 10.00% S/L ratio and 15.0 h) is aligned with some author’s suggestion for process sustainability, in terms of costs. They indicate that the presence of 10 to 20% of the non-hydrolyzed substrate in the reaction medium might be a more effective strategy (Arantes & Saddler, 2011; Shen & Agblevor, 2008).

Asada et al. (2015) reported a saccharification slightly higher than what is seen in this paper, with a glucose yield of 89% for pre-treated Beechwood using enzymatic hydrolysis (initial substrate concentration of 2%, 0.1 g of enzyme per 1 g of the substrate, 120, 50 °C). Alrumman (2016) described an enzymatic saccharification yield of 71.03% with *Geobacillus stearothermophilus* palm waste (4% S/L ratio, 30 FPU/g enzyme, 5.0 pH, 50 °C, 24 h).

It is possible to observe in the fermentation experiment with *Saccharomyces cerevisiae* that 28.99% of the reducing sugars present in the medium were not consumed. The pretreatment processes of lignocellulosic biomasses employed in this work are widely used strategies; however, they have the disadvantage of generating toxic compounds to fermenting organisms, such as phenolic compounds, guaiacol, levulinic acid, furfural and 5-hydroxymethylfurfural (Varanasi et al., 2013; Liu et al., 2016). Although the pre-treated endocarp was rinsed with distillate water before enzymatic scarification, Zhang et al. (2010) indicate that this method is not efficient for total removal of toxic compounds impregnated in the biomass. Thereby, the presence of such substances may have inhibited the activity of *S. cerevisiae*, making it impossible to deplete the substrate offered.

Kamoldeen et al. (2017), Koti et al. (2016) and Jing-Ping et al. (2011) reported similar ethanol yields in fermentations of oil palm empty fruit bunches with *Saccharomyces cerevisiae* ATCC 24858 (0.33 g g^−1^), wheat straw with *Pichia stipitis* PSEB5 (0.34 g g^−1^) and corncob using *Candida shehatae* ACCC 20335 (0.31 g g^−1^), respectively. However, the ethanol yield (Y_P∕S_) achieved by Ko et al. (2016) using rice straw hydrolysate reached 0.46 g g^−1^, a value significantly higher than what is detected in this study.

## Conclusion

Buriti fruit endocarps were successfully converted to bioethanol by *Saccharomyces cerevisiae*. The sequential use of diluted acid (10% S/L ratio, 120 °C, 7% H_2_SO, 20 min) and alkaline (10% S/L ratio, 80 °C, 12% NaOH, 48 h) hydrolysis, under optimized conditions, contributed significantly to the reduction of hemicellulose and lignin contents in the pre-treated biomass. These treatments reverberated in the enzymatic saccharification step, in which 86% of the cellulose was converted to glucose in optimal conditions (10% S/L ratio, 50 °C, 60 µL g^−1^ cellulase, 15 h). The efficiency of the fermentative process (43.16 g L^−1^ of ethanol, 77% efficiency) was comparable with literature descriptions of ethanol production using lignocellulosic substrates. Furthermore, the execution of a fermentative process with a higher degree of control, and the detoxification of the saccharified buriti endocarp may contribute to enhance ethanol yield and viability.

##  Supplemental Information

10.7717/peerj.5275/supp-1 Data S1 Raw data: endocarp saccharification

10.7717/peerj.5275/supp-2 Data S2 Raw data: acid pretreatment

10.7717/peerj.5275/supp-3 Data S3 Raw data: alkali treatment

## Additional Information and Declarations

### Data Availability

The following information was supplied regarding data availability: The raw data are included in the Supplemental Files.
